# Epistemicide, health systems, and planetary health: Re-centering Indigenous knowledge systems

**DOI:** 10.1371/journal.pgph.0003634

**Published:** 2024-08-29

**Authors:** Nicole Redvers, Amali U. Lokugamage, João Paulo Lima Barreto, Madhu Bajra Bajracharya, Matthew Harris

**Affiliations:** 1 Schulich School of Medicine and Dentistry, University of Western Ontario, London, Canada; 2 Arctic Indigenous Wellness Foundation, Yellowknife, Canada; 3 Institute of Women’s Health, University College London, London, United Kingdom; 4 Whittington Hospital NHS Trust, London, United Kingdom; 5 Federal University of Amazonas, Manaus, Brazil; 6 Piyushabarshi Aushadhalaya, Kathmandu, Nepal; 7 Department of Primary Care and Public Health, School of Public Health, Imperial College London, London, United Kingdom; PLOS: Public Library of Science, UNITED STATES OF AMERICA

The assumed dominance of Euro-Western thinking in current biomedical structures is intimately related to colonial expansion, and has been consolidated through institutions such as universities, who have often situated themselves as the colonial matrices of power [1]. Present-day biomedical structures within health systems (including within medical journal structures) have continued to evolve from past and present colonial eras while carrying with it Euro-Western colonial biases and prejudices [2]. Colonial imperialism and the expansion of territories, power, and the extraction of resources from those colonized, was also accompanied by imposed Westernized epistemological (knowledge) dominance—the often-violent steamrolling of Western ideas over the ideas of those forcibly colonized [1]. Given this, some of the ongoing effects of colonization are the deeply embedded structural power imbalances that continue to cement the domination of the colonizers and their knowledge systems [3]. This assumed Western knowledge domination was purposeful, as a plurality of ideas would have undermined colonialism itself.

Ideological imperialism subjugated and marginalized epistemological systems of colonized communities and Nations, resulting in”epistemicide” [4]. “Epistemicide” itself is the killing, silencing, annihilation, or devaluing of a knowledge system [4]. Epistemic violence has been mediated through colonial power imbalances so that white, patriarchal, abled-bodied people have been enabled to create the vision of normality while exerting hegemonic dominance and oppression on those who do not ‘fit the mold’ [1]—implicitly or explicitly. Epistemicide is currently an ongoing phenomenon with The International Work Group for Indigenous Affairs stating that, “[f]or five centuries, a systematic attack has persisted in a bid to bring an end to the creation, conservation, and transmission of the knowledge of [Indigenous] Peoples [5].

Epistemicide ensures that knowledge outside the Euro-Western sphere is discredited while adding credibility and uplifting Euro-centric values and knowledge traditions—implicitly or explicitly [6]. Past and ongoing colonialism and consequent epistemicide additionally continues to uplift racism, sexism, anti-2SLGBTQ+ prejudice, ableism, and classism, which creates barriers to effectively addressing health inequities. Euro-Western centricity additionally ensures that power is concentrated in the hands of often white cis-patriarchal males which perpetuates continued intersectional disadvantage to marginalized people and communities. Ideological imperialism has therefore left a profound imprint, a structural legacy on current knowledge systems including healthcare structures, which contributes to morbidity and mortality within marginalized communities [7].

Decolonizing healthcare movements have increasingly been mobilized to overturn enduring colonial injustices that remain entangled within the structures of Euro-Western-centric health systems [2,7]. Substantial barriers continue to exist, however, which mandates an honest self-reflection on how health systems continue to enable epistemicide. Attaining a ‘decolonial future’ is an urgent need to forge a fairer future while enabling the dismantling of pernicious biases and prejudices that persist in the structures of healthcare delivery around the globe.

## Epistemicide and Indigenous knowledge systems

Within the Indigenous context, “nothing about us, without us” platforms the need for appropriate author positionality within this article as is increasingly being called for from within Indigenous Nations [8]. Given this, NR, JPLB, and MBB position themselves from Indigenous communities located in Canada, Brazil, and Nepal respectively. AUL positions herself as a South Asian decolonial scholar based in the United Kingdom (UK) but originates from Sri Lanka, and MH positions himself as a public health scholar and physician based in the UK with strong connections to Brazil.

Euro-Western scientific systems, which healthcare systems are based on, have embodied a spirit of scientific hegemony that has pervaded most branches of clinical practice, research, and inquiry. This scientific hegemony has been at the expense of Indigenous knowledge systems. Indigenous knowledge systems are systems of knowledge, know-how, skills, and practices that are developed, sustained, and passed on from generation to generation within a community, often forming part of its cultural or spiritual identity [9]. The assumed superiority generally of Euro-Western-centric knowledge systems specifically over Indigenous knowledge systems has been a continued and ongoing legacy of colonialism around the globe. With this, knowledge democracy [10] for Indigenous Peoples has not prevailed.

Given this, before addressing issues of health equity, there must be an appreciation and understanding for how even ways of knowing, ways of being in the world, and ways of carrying out inquiry are also steeped in considerations of social justice and the mere democracy of knowledge. Lakota physician, Dr. Donald Warne, aptly states that “if we’re ever going to achieve equity across populations…we have to walk through truth” [11].^.^ Walking through truth is not an easy process; however, it is necessary to address decades of epistemicide that has further perpetuated harm within Indigenous communities.

## Epistemicide and planetary health

Indigenous knowledge systems are process-based and applied, with every lived experience within Indigenous communities constituting knowledge in action. Additionally, as Indigenous knowledges are directly connected to Land in the all-encompassing sense (i.e., Land, water, air, living things) [12], the health and wellbeing of Indigenous communities is therefore completely and utterly interconnected to the health of Land. One of the consequences of the attempted epistemicide of Indigenous knowledges through colonization has been the forced disconnection between health systems and Nature (i.e., the Land) [13]. For example, heath systems worldwide are currently one of the largest contributors to greenhouse gas emission, with emissions higher than that of the aviation industry [14].

On the wider scale, through the assumed dominance of Euro-Western-centric worldviews, most societies consider themselves to be entirely separate from Nature, with their health dependent only on their access to healthcare and a range of favorable social determinant conditions. This view persists despite all socioeconomic and health inputs being completely dependent on a healthy and livable planet. Euro-Western-centric health systems and the Nature disconnected connotations of health they have perpetuated are themselves in ecological denial. Due to this health system ecological denial, perpetuated by ongoing epistemicide, all of humanity continues to be at risk from Nature-disconnected systems and therefore current realities such as climate change.

On the flip side, from the perspective of numerous Indigenous cosmologies, in order to heal the world, the health of the planet is just as important as healing individuals and communities [15]. Indigenous health systems value all aspects of Nature (e.g., earth, fire, wind, water, rocks, plant, and animal relatives), and have deep reverence for the patterns and interconnectedness of Nature across Mother Earth and the greater universe [16]. Many Indigenous views of health and wellbeing therefore continue to center holistic understandings of planetary health, whereas biomedicine rarely takes into consideration the interlinking effects of fields such as human biology and ecology [17]. With this, the Euro-Western-centric worldview has perpetuated ecological erasure and therefore our own human demise.

The recognition of this ecological erasure in how we currently approach health has led to a surge in calls for Indigenous Planetary Health and its direct connection to human wellbeing to be included in health policy dialogues [18]. This re-centering of Indigenous Peoples and their knowledges around planetary health as being central to human wellbeing [15] has only started to be appreciated by health systems in a time of coalescing global crises. It is well known within biodiversity circles that ecosystems are more resilient when they are diverse and heterogenous.

Indigenous Peoples appreciate that this need for “diversity” is also applied to knowledge systems to build resiliency. When you homogenize knowledge, as has been done through ongoing epistemicide, our resilience as a human species declines just like a monoculture farm starts to lose its ability to buffer stresses from environmental changes. Epistemicide has therefore resulted in decreased resilience and wellbeing as a human society when looked at from an Indigenous worldview.

We require the “gift of multiple perspectives” [19] to withstand the increasing dysfunction brought about by Euro-Western-centric worldviews that have led us to the precipice of ecological genocide. With this, the healthcare enterprise including medical journal publishing enterprises have to take key responsibility for the role it has played in epistemicide and the consequent devaluing of Indigenous Peoples and Nature (see *Box 1*). There is a need for the healthcare and medical journal enterprises to walk through truth while amplifying the path toward Indigenous and ecological reconciliation [20] (see *Table 1* for example recommendations for re-centring decolonial knowledges within medical publishing).

**Box 1. Key messages for health systems**

- Epistemicide is the killing, silencing, annihilation, or devaluing of a knowledge system. Euro-Western knowledge domination was purposeful, as a plurality of ideas would have undermined colonialism itself.
- One of the consequences of the attempted epistemicide of Indigenous knowledge systems through colonization has been the forced disconnection between health systems and Nature.
- When you homogenize knowledge, as has been done through ongoing epistemicide, our resilience as a human species declines just like a monoculture farm starts to lose its ability to buffer stresses from environmental changes. Diversity of knowledge is important.
- Reversing the lingering colonial epistemicide of the ecological knowledge base of Indigenous health systems are vital for the healing of individuals, communities, and the planet.

**Table 1:** Recommendations for the re-centring decolonial knowledges, and therefore Land-centric knowledges within medical publishing.

1	Publishing companies to increase, and actively support through appropriate payment models, editors from historically and contemporarily marginalized backgrounds and contexts including from the Global South and from Indigenous communities globally.
2	Editorial teams to exercise transparent reflexivity, with public facing accountability mechanisms in place, on who is vetting articles, who is controlling knowledge processes (including dealing with Euro-centric peer review bias), throughout the entire publication process.
3	Academic journals to adopt and commit to cultural safety education and practice across all elements of the publication process which requires special attention to elements of power across the publishing continuum.
4	Academic journals to instigate and action community engagement around publishing priorities that matter to historically and contemporarily marginalized communities.
5	Academic journals to create explicit policies to require positionality statements for all authors (see *Roach et al*, *2023* [8]), in addition to requirements for specifying how the affected community was involved in all stages of the research process (including being named as authors) when carrying out research with marginalized peoples and communities.
6	Academic journals to invite more editorials and publications from those who have been marginalized by colonisation with waived article process fees and a commitment to open access
7	Publishing companies with the support of academic journals to create more equitable mechanisms for supporting non-English language authors and publications.

Platforming and making space for non-Euro-Westernized knowledges requires the ongoing walking through the truth—colonialism and epistemicide has harmed the health of the planet and the health of all. Disrupting and dismantling Euro-Western-centric systems of knowledge that have mobilized top-down power structures for centuries will facilitate a decolonial future with more favorable health outcomes for all—including the planet.
